# Efficacy of Liver Resection for Single Large Hepatocellular Carcinoma in Child-Pugh A Cirrhosis: Analysis of a Nationwide Cancer Registry Database

**DOI:** 10.3389/fonc.2021.674603

**Published:** 2021-04-30

**Authors:** Suk Kyun Hong, Kwang-Woong Lee, Su young Hong, Sanggyun Suh, Kwangpyo Hong, Eui Soo Han, Jeong-Moo Lee, YoungRok Choi, Nam-Joon Yi, Kyung-Suk Suh

**Affiliations:** Department of Surgery, Seoul National University College of Medicine, Seoul, South Korea

**Keywords:** hepatocellular carcinoma, hepatectomy, prognosis, survival, recurrence

## Abstract

**Background:**

Therapeutic strategies and good prognostic factors are important for patients with single large hepatocellular carcinoma (HCC). This retrospective study aimed to identify the prognostic factors in patients with single large HCC with good performance status and Child-Pugh A cirrhosis using a large national cancer registry database and to recommend therapeutic strategies.

**Methods:**

Among 12139 HCC patients registered at the Korean Primary Liver Cancer Registry between 2008 and 2015, single large (≥ 5 cm) HCC patients with Eastern Cooperative Oncology Group (ECOG) performance status 0 and Child-Pugh score A were selected.

**Results:**

Overall, 466 patients were analyzed. The 1-,2-,3-, and 5-year survival rates after initial treatment were 84.9%, 71.0%, 60.1%, and 51.6%, respectively, and progression-free survival rates were 43.6%, 33.0%, 29.0%, and 26.8%, respectively. Platelet count < 100 × 10^9^/L (*P* < 0.001), sodium level < 135 mmol/L (*P* = 0.002), maximum tumor diameter ≥ 10 cm (*P* = 0.001), and treatment other than resection (transarterial therapy vs. resection: *P* < 0.001, others vs. resection: *P* = 0.002) were significantly associated with poorer overall survival; sodium < 135 mmol/L (*P* = 0.015), maximum tumor diameter ≥ 10 cm (*P* < 0.001), and treatment other than resection (transarterial therapy vs. resection: *P* < 0.001, others vs. resection: *P* = 0.001) were independently associated with poorer progression-free survival.

**Conclusion:**

Resection as an initial treatment should be considered when possible, even in patients with single large HCC with good performance status and mild cirrhosis. Caution should be exercised in patients with low platelet level (< 100 × 10^9^/L), low serum sodium level (< 135 mmol/L), and maximum tumor diameter ≥ 10 cm.

## Introduction

Surveillance programs for detecting hepatocellular carcinoma (HCC) vary with each country depending on the prevalence of HCC. Since hepatitis B virus is endemic to Korea and Japan, the prevalence of HCC is high. The recommended surveillance programs for HCC detection include liver ultrasonography and monitoring serum alpha-fetoprotein (AFP) levels (1, 2). However, despite surveillance programs, the proportion of diagnosis of large HCC is still substantial (3). When treating HCC, tumor factors including number, size, and aggressiveness, together with underlying liver function and performance status should be considered. Liver resection can be considered as the first-line treatment in patients with single HCC confined to the liver without cirrhosis in cases where the residual liver function is expected to be sufficient even with cirrhosis (4–6). The most recent version of the combined American Joint Committee on Cancer (AJCC)/Union for International Cancer Control (UICC) TNM staging system from 2017 states that patients with multiple tumors, any of which are > 5 cm, are categorized as T3 (7). Previous studies have reported that although the 5-year survival was similar in patients with and without cirrhosis who had single HCC ≤ 5 cm, it was worse in patients with cirrhosis with HCC > 5 cm (8). However, recent studies demonstrated that microvascular invasion was not observed in about one-third of patients with tumor size > 10 cm and positive outcomes were reported after resection in these patients; thus, resection should not be considered based on solely the size of the tumor (3, 9). However, these studies had limitations, such as small sample sizes, lack of comparison groups, and/or selection bias. Moreover, treatment for single large (≥ 5 cm) HCCs is still debated, with limited data available.

This study aimed to identify prognostic factors for single large HCC using a large, nationwide cancer registry database. To focus on the effectiveness of resection and to minimize selection bias, we restricted the cohort to patients with Child-Pugh class A and an Eastern Cooperative Oncology Group (ECOG) PERFORMANCE STATUS OF 0.

## Materials and Methods

### Patients and Methods

The present study was approved by the Institutional Review Board of the Seoul National University Hospital (IRB No. 2101-088-1189). Due to the retrospective nature of the study, the requirement for patient consent was waived. The study population was obtained from the Korean Primary Liver Cancer Registry (KPLCR), which represents a national, random sample of approximately 15% of patients registered in the Korean Central Cancer Registry (KCCR). Considering that KCCR accounts for more than 95% of all cancer cases in Korea, the KPLCR represents a group of patients with newly diagnosed HCC. The following data were obtained from the KPLCR database: age, sex, height, weight, smoking history, alcohol history, medical history of diabetes and hypertension, underlying liver disease, performance status, Child-Pugh score, Model for End-Stage Liver Disease (MELD) score, laboratory results, including AFP and proteins induced by vitamin K absence-II (PIVKA-II), diagnosis date, tumor number, maximum size, macrovascular invasion, distant metastasis, initial treatment modality and date, secondary treatment modality and date, and survival outcome.

**Figure 1:**
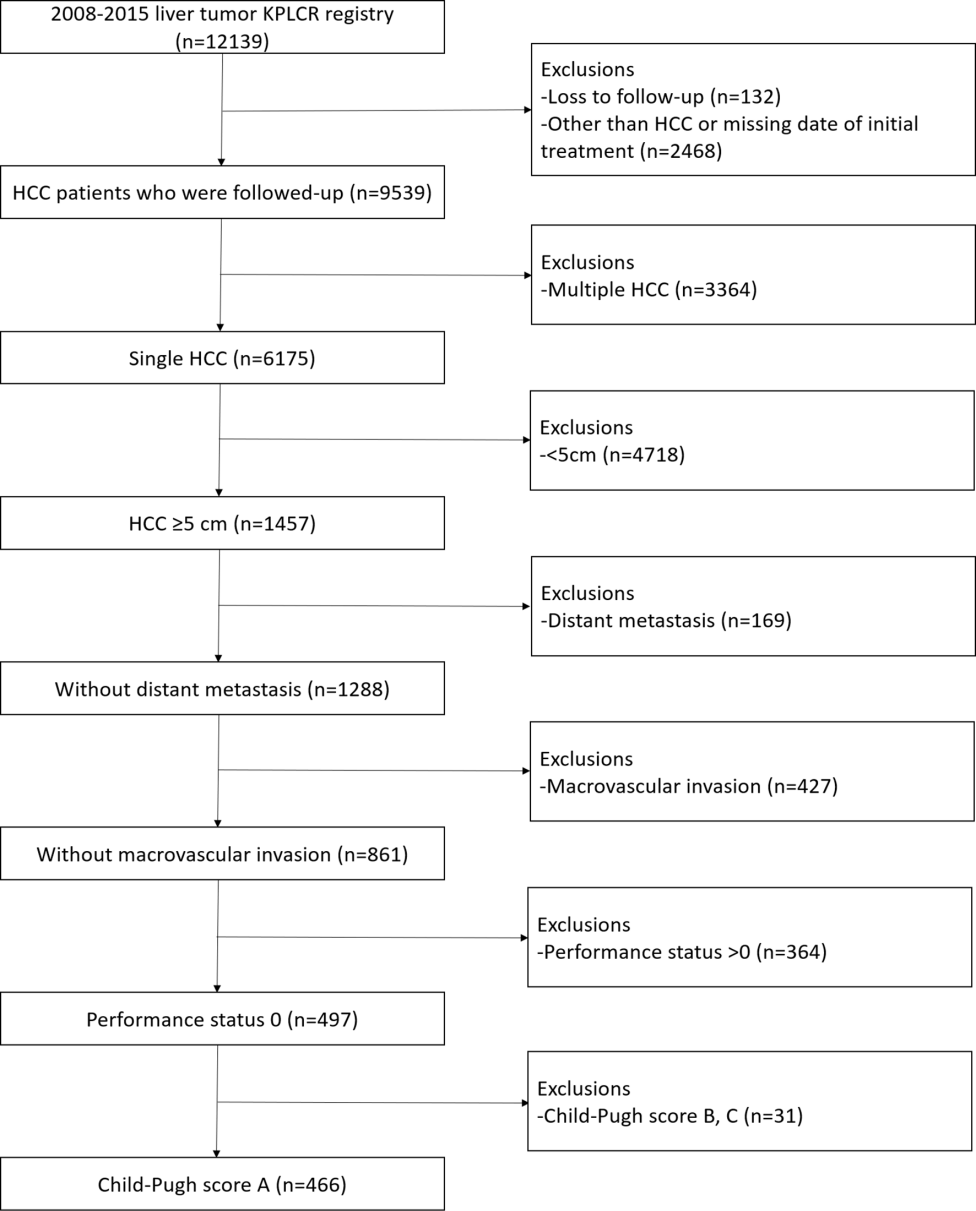
Flowsheet of the enrolled patients.

**Figure 1** shows the flowchart for the selection of study population. Between 2008 and 2015, 12139 liver tumor patients were registered in the KPLCR from 54 hospitals. The potential cohort included all the patients registered under KPLCR, except those without a follow-up or missing treatment dates. Of these, 1457 patients had single large (≥ 5 cm) HCCs. Patients with distant metastasis or macrovascular invasion were excluded from the study. Furthermore, patients with ECOG performance status other than 0 and Child-Pugh grade other than grade A were also excluded. Finally, 466 patients were included in the study. The duration of survival was measured from the date of initial treatment to the date of death or the last follow-up date. The duration of progression-free survival was measured from the initial treatment date to the second or last follow-up date. The cut-off values of continuous variables were selected considering previous publications.

### Statistical Analysis

Results are expressed as mean ± standard deviation or median (range) for continuous data and as numbers with percentages for categorical data. Survival and progression-free survival were calculated using the Kaplan-Meier method and compared using the log-rank test. Multivariate Cox proportional hazards regression with backward selection was used to determine the effect of statistically significant variables in the univariate analysis. A *P*-value of < 0.05 was considered significant. Statistical analysis was performed using the SPSS software (version 25; SPSS Inc., Chicago, IL, USA).

## Results

### Baseline Characteristics

There were 466 HCC patients with single and large (≥ 5 cm) tumors with Child A, performance status of 0, but without distant metastasis and macrovascular invasion. **Table 1** summarizes the demographic characteristics of these patients. The mean age was 61.3 years, and the mean body mass index (BMI) was 24.0 kg/m^2^. More than half of the patients (56.0%) tested positive for hepatitis B surface antigen. The mean Child-Pugh score was 5.2, whereas all the patients were Child grade A according to the selection criteria of this study. The mean platelet count was 201.9 × 10^9^/L, and the mean total bilirubin level was 0.8 mg/dL. The median serum AFP was 34.6 ng/mL (0.4-200000.0) and median PIVKA-II was 936.0 mAU/mL (5.0- 95926.0). The most common initial treatment modality was resection (52.8%), followed by transarterial therapy, most of which was transarterial chemoembolization (TACE) (43.8%).

**Table 1:** Demographic and clinical characteristics of the included patients.

Variables	N = 466
Demographic variables	
Age (year)	61.3 ± 11.9
Male: Female	388: 78
BMI (kg/m^2^)	24.0 ± 3.5
Smoking	214 (45.9)
Alcohol	149 (32.0)
Diabetes	118 (25.3)
Hypertension	184 (39.5)
Etiology	
HBV^*^	261 (56.0)
HCV^*^	40 (8.6)
Non-B Non-C	156 (33.5)
Child-Pugh score	5.2 ± 0.4
MELD score	8.0 ± 2.0
Laboratory variable	
Platelet count (10^9^/L)	201.9 ± 85.3
Total bilirubin (mg/dL)	0.8 ± 0.4
Serum albumin (g/dL)	4.1 ± 0.5
Alanine aminotransferase (IU/L)	48.1 ± 55.7
Prothrombin time (INR)	1.1 ± 0.1
Creatinine (mg/dL)	1.0 ± 0.8
Sodium (mmol/L)	139.6 ± 3.2
Alpha-fetoprotein (ng/mL)	34.6 (0.4-200000.0)
PIVKA-II (mAU/mL)	936.0 (5.0- 95926.0)
Tumor variable	
Maximum tumor diameter (cm)	8.0 ± 3.0
Treatment variable	
Initial treatment	
Resection	246 (52.8)
Transarterial therapy	204 (43.8)
Chemotherapy	8 (1.7)
Local ablative therapy	3 (0.6)
Radiation	3 (0.6)
Liver transplantation	2 (0.4)

*Four patients had underlying hepatitis B and C coinfections.

BMI, body mass index; HBV, hepatitis B virus; HCV, hepatitis C virus; MELD, Model for End-Stage Liver Disease; PIVKA-II, protein induced by vitamin K absence-II.

### Overall Survival Rates and Progression-Free Survival Rates

**Figure 2:**
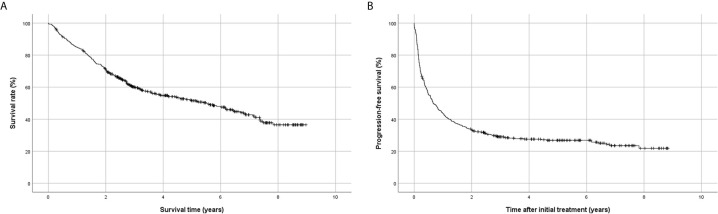
Kaplan-Meier analysis of survival using the Korean Primary Liver Cancer Registry database. **(A)** Overall survival, **(B)** progression-free survival.

Kaplan-Meier analysis showed that the 1-,2-,3-, and 5-year survival rates after initial treatment were 84.9%, 71.0%, 60.1%, and 51.6%, respectively (**Figure 2A**), and the 1-,2-,3-, and 5-year progression-free survival rates were 43.6%, 33.0%, 29.0%, and 26.8%, respectively (**Figure 2B**). The 1-,2-,3-, and 5-year survival rates after resection were 92.6%, 81.1%, 73.7%, and 65.6%, and the 1-,2-,3-, and 5-year progression-free survival rates were 62.8%, 50.0%, 45.0%, and 43.2%, respectively.

### Factors Affecting Overall Survival and Progression-Free Survival

**Figure 3:**
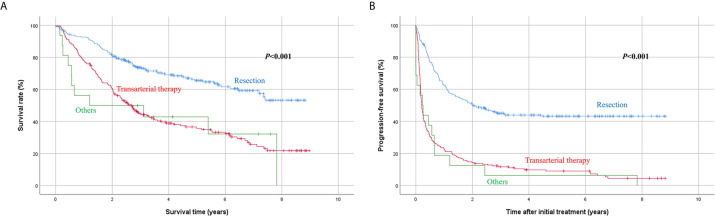
Kaplan-Meier analysis of survival according to initial treatment modality. **(A)** Overall survival, **(B)** progression-free survival.

Univariate analysis showed that the Child-Pugh score, MELD score, platelet count, total bilirubin, serum albumin, sodium, maximum tumor diameter, and initial treatment modality were associated with survival rate (**Table 2**). Among these factors, platelet count < 100 × 10^9^/L (*P* < 0.001), sodium level < 135 mmol/L (*P* = 0.002), maximum tumor diameter ≥ 10 cm (*P* = 0.001), and treatment other than resection (transarterial therapy vs. resection, *P* < 0.001; others vs. resection, *P* = 0.002) (**Figure 3A**) were significantly associated with poorer overall survival.

**Table 2:** Univariate and multivariate analyses of factors affecting overall survival and progression-free survival.

Demographic variables			N = 466	Overall survival				Progression-free survival			
				Univariate	Multivariate			Univariate	Multivariate		
				*P*-value	HR	95% CI	P-value	*P*-value	HR	95% CI	*P*-value
Age (year)											
	<60		203								
	≥60		263	0.233				0.186			
Sex											
	Male		388								
	Female		78	0.091				0.500			
BMI (kg/m^2^)											
	<25		296								
	≥25		159	0.216				0.059			
Smoking											
	No		251								
	Yes		214	0.760				0.058			
Alcohol											
	No		311								
	Yes		149	0.990				0.288			
Diabetes											
	No		348								
	Yes		118	0.859				0.275			
Hypertension											
	No		282								
	Yes		184	0.588				0.480			
Etiology											
	HBV										
		No	195								
		Yes	261	0.481				0.312			
	HCV										
		No	400						Reference		
		Yes	40	0.058				0.037	–	–	0.533
Child-Pugh score											
	5		368		Reference				Reference		
	6		98	<0.001	–	–	0.155	0.001	–	–	0.214
MELD score											
	<9		333		Reference						
	≥9		128	0.033	–	–	0.165	0.254			
Laboratory variables											
Platelet count (10^9^/L)											
	<100		40		Reference				Reference		
	≥100		425	<0.001	0.452	0.306-0.668	<0.001	<0.001	–	–	0.484
Total bilirubin (mg/dL)											
	<1.2		386		Reference						
	≥1.2		50	0.010	–	–	0.248	0.242			
Serum albumin (g/dL)											
	<3.5		54		Reference				Reference		
	≥3.5		412	0.008	–	–	0.561	0.007	–	–	0.101
Alanine aminotransferase (IU/L)											
	<50		338								
	≥50		127	0.845				0.482			
Prothrombin time (INR)											
	<1.2		429		Reference						
	≥1.2		37	0.003	–	–	0.162	0.308			
Creatinine (mg/dL)											
	<1.0		300								
	≥1.0		164	0.607				0.721			
Sodium (mmol/L)											
	<135		24		Reference				Reference		
	≥135		438	<0.001	0.468	0.292-0.750	0.002	<0.001	0.477	0.263-0.864	0.015
Alpha-fetoprotein (ng/mL)											
	<1000		343								
	≥1000		102	0.075				0.604			
PIVKA-II (mAU/mL)											
	<1000		171						Reference		
	≥1000		166	0.450				0.008	–	–	0.118
Tumor variables											
Maximum tumor diameter (cm)											
	<10		367		Reference				Reference		
	≥10		99	0.005	1.693	1.243-2.306	0.001	<0.001	1.891	1.369-2.611	<0.001
Treatment variable											
Initial treatment				<0.001				<0.001			
	Resection		246		Reference				Reference		
	Transarterial therapy		204		2.167	1.623-2.893	<0.001		3.736	2.821-4.949	<0.001
	Others		16		2.660	1.413-5.006	0.002		3.113	1.591-6.091	0.001

BMI, body mass index; CI, confidence interval; HBV, hepatitis B virus; HCV, hepatitis C virus; HR, hazard ratio; MELD, Model for End-Stage Liver Disease; PIVKA-II, protein induced by vitamin K absence-II.

Another univariate analysis identified that hepatitis C virus infection as an underlying liver disease (*P* = 0.037), Child-Pugh score 6 (*P* = 0.001), platelet count < 100 × 10^9^/L (*P* < 0.001), serum albumin < 3.5 g/dL (*P* = 0.007), sodium level < 135 mmol/L (*P* < 0.001), PIVKA-II level ≥ 1000 mAU/mL (*P* = 0.008), maximum tumor diameter ≥ 10 cm (*P* < 0.001), and initial treatment other than resection (*P* < 0.001) were associated with poorer progression-free survival after initial treatment. Multivariate analysis revealed that sodium < 135 mmol/L (*P* = 0.015), maximum tumor diameter ≥ 10 cm (*P* < 0.001), and treatment other than resection (transarterial therapy vs. resection, *P* < 0.001; others vs. resection, *P* = 0.001) (**Figure 3B**) were independently associated with poorer progression-free survival.

## Discussion

Liver resection is widely recognized as the first-line treatment in patients with HCC when feasible, considering patient performance status, remnant liver function, and tumor factors (4–6). According to previous studies, liver resection in Child A patients showed good results, with an average 5-year survival of over 60% and long-term intrahepatic control rates of over 40% (10, 11). The patients in our study had large HCC with performance status 0 and Child A cirrhosis. Among them, 246 (52.8%) patients underwent resection, and showed similar 5-year survival (65.6%) and progression-free survival (43.2%) rates. This is in line with previous studies that demonstrated comparable outcomes in large HCC patients and proposed considering resection not based solely on tumor size (3, 9). Lin et al. reported that Barcelona Clinic Liver Cancer (BCLC) stage B, Child A showed better survival rates in patients who underwent resection than in those who underwent TACE (12). Several other recent studies showed survival benefit of resection for BCLC stage B HCC (13–19). Hwang et al. demonstrated that resection followed by active recurrence treatment improved survival even in patients with HCC ≥ 10 cm (3). Multivariate analysis in our study also showed that resection was associated with significantly better survival and progression-free survival than other treatments, most of which were transarterial therapy.

Although the outcomes of patients with single large (≥ 5 cm) HCC who underwent resection as the initial treatment were comparable with previous reports, multivariate analysis demonstrated that a maximum tumor diameter ≥ 10 cm showed poorer overall survival and progression-free survival than a maximum tumor diameter < 10 cm. A recent study also reported poorer 5-year overall survival and recurrence-free survival in patients with huge HCC (≥ 10 cm) than in patients with HCC < 10 cm and identified tumor size ≥ 10 cm as an independent risk factor of initial extrahepatic recurrence. Although this study does not contain any information on the location or pattern of recurrence, it can be roughly inferred from the second treatment. Out of the 466 patients, 266 (57.1%) underwent a second treatment due to disease progression. Of the 266 patients, more than half underwent transarterial treatment (64.7%) as the second treatment, followed by local ablation (10.5%), chemotherapy (10.2%), resection (9.8%), and radiation (4.9%). Considering the increased proportion of chemotherapy and radiation in the second treatment compared to the initial treatment, disease progression in our study may not be limited to the liver.

**Figure 4:**
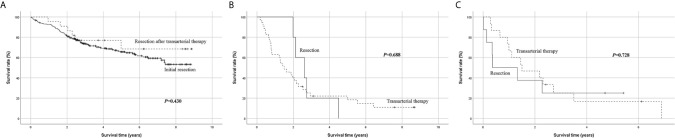
Subgroup Kaplan-Meier analysis of overall survival. **(A)** Initial resection vs. resection after transarterial therapy, **(B)** resection vs. transarterial therapy in patients with low platelet count < 100 × 10^9^/L, **(C)** resection vs. transarterial therapy in patients with low serum sodium < 135 mmol/L.

Of the 204 patients who underwent transarterial treatment as initial treatment, 22 underwent resection as a secondary treatment. Considering that 13 of the 22 patients underwent resection after transarterial treatment within 2 months, these patients may have been intentionally treated with TACE before surgery. Their overall survival was similar to that of patients who underwent resection as initial treatment (*P* = 0.430) (**Figure 4A**), suggesting that resection after transarterial therapy can be another possible therapeutic option for patients with single large HCC with tolerable liver function. This finding was similar to that of other studies that show that resection after TACE may be considered as an effective method in some intermediate-stage HCC patients (20, 21).

Two patients underwent liver transplantation (LT) as initial treatment. One patient with a maximum tumor diameter of 8 cm, AFP level of 5.3 ng/mL, and PIVKA-II 500 mAU/mL survived 7.8 years after LT without recurrence until death. Another patient with a maximum tumor diameter of 12 cm, AFP level of 18 ng/mL, and PIVKA-II 19 mAU/mL died 1.2 years after LT without recurrence until death. These patients must have undergone LT as per the Milan criteria based on biologic markers, such as AFP and PIVKA-II (22). However, due to the small number of patients, the efficacy of LT in single large HCC should be further studied using the national LT database.

Several studies have reported the association between preoperative low platelet count and poor prognosis in different kinds of cancer, including HCC (23, 24). Preoperative thrombocytopenia was reported to be associated with overall survival as well as recurrence-free survival in HCC patients (25–28). Although the exact reason for poor outcome in HCC with low platelet count needs to be studied further, one of the possible explanations could be the frequent occurrence of thrombocytopenia in cirrhotic patients with portal hypertension (29–32). Portal hypertension reflects the severity of cirrhosis, which is a well-known risk factor related to late recurrence in patients with HCC. The mechanism of late recurrence can be explained by multicentric recurrence in the remnant liver (31, 32). Regarding surgical resection, liver fibrosis and significant portal hypertension are risk factors for postoperative hepatic decompensation, which is a serious complication after resection (30). According to our study, multivariate analysis revealed that preoperative low platelet counts significantly affected poor overall survival. Although the cohort in the study included only patients with Child A, the low platelet count among these patients still affected poor survival. However, even though a low platelet count was associated with poor progression-free survival in the univariate analysis, it was not statistically significant in the multivariate analysis.

Hyponatremia due to splanchnic vasodilatation, which reduces the effective circulating blood volume, is frequently seen in cirrhotic patients (33–36). Serum sodium levels tend to decrease as liver cirrhosis progresses (34–36). Several studies have previously reported the prognostic role of serum sodium levels in patients with HCC (33, 37). Min et al. reported that sodium levels were an independent risk factor for post-TACE acute hepatic failure (38). Nishikawa et al. analyzed 1170 HCC patients with liver cirrhosis and revealed that serum sodium level was independently associated with overall survival (33). According to their study, 804 patients had Child A cirrhosis and 41 of them (5.1%) had serum sodium levels < 135 mmol/L. In the present study, which included only Child A patients, 24 (5.2%) patients had hyponatremia (< 135 mmol/L). Similar to previous reports, the present study also revealed that serum sodium levels < 135 mmol/L were independently associated with poor overall survival and progression-free survival.

When performing subgroup analysis limited to patients with platelet count < 100 × 10^9^/L, there was no significant difference in survival between resection and transarterial therapy as initial treatment (*P* = 0.688) (**Figure 4B**). Another subgroup analysis limited to patients with serum sodium < 135 mmol/L also showed no significant survival difference between resection and transarterial therapy as initial treatment (*P* = 0.728) (**Figure 4C**). The survival benefit of resection disappeared when the patients had a low platelet count < 100 × 10^9^/L or serum sodium level < 135 mmol/L.

This study has several limitations that should be considered. First, it was a retrospective study using a national registry database that relied on the completeness of medical records. There are risks of data loss and differences between centers with regard to data recording. However, the patients in the KPLCR were selected from the KCCR using the probability proportional to size method to minimize selection bias, and thus became representative of all Korean HCC patients. Second, in some instances, relevant data, including comorbidities other than smoking, alcohol history, and complications after treatment were missing. Third, the database contains the date of initial treatment and the date of the second treatment, which allowed the calculation of disease progression-free survival. However, recurrence-free survival could not be assessed because second treatment does not entail recurrence. Moreover, data for progression-free survival may be biased since the second treatment date or the date of death as reference for progression can be inappropriate and inaccurate considering the nationwide retrospective nature of this study. Despite these limitations, a notable strength of this study is that it is a large study using a national registry database focused on single large HCC patients with performance status 0 and Child A. There are only few studies with sufficient sample size that have been analyzed in this particular patient group.

The outcomes of single large HCC patients with Child A were satisfactory, which is consistent with the results of previous worldwide studies. Resection should be initially considered whenever possible, and not dismissed based only on tumor size. Patients with low serum sodium levels (< 135 mol/L) and HCCs ≥ 10 cm had a higher risk of poor overall survival and disease progression-free survival.

## Data Availability Statement 

The original contributions presented in the study are included in the article/supplementary material. Further inquiries can be directed to the corresponding author.

## Ethics Statement

The studies involving human participants were reviewed and approved by Institutional Review Board of the Seoul National University Hospital (IRB No. 2101-088-1189). Written informed consent from the participants’ legal guardian/next of kin was not required to participate in this study in accordance with the national legislation and the institutional requirements. Written informed consent was not obtained from the minor(s)’ legal guardian/next of kin for the publication of any potentially identifiable images or data included in this article.

## Author Contributions

SKH and K-WL made the concept and design. SKH, K-WL, SYH, and SS acquired, analyzed, and interpreted the data. SKH drafted the manuscript. SKH, K-WL, SYH, SS, KH, EH, J-ML, YC, NY, and K-SS critically revised the manuscript for important intellectual content. All authors contributed to the article and approved the submitted version.

## Conflict of Interest

The authors declare that the research was conducted in the absence of any commercial or financial relationships that could be construed as a potential conflict of interest.
